# Small RNA sequencing identifies tsRNA-05020 as a potential regulator of cervical cancer progression

**DOI:** 10.55730/1300-0152.2802

**Published:** 2026-02-03

**Authors:** Cheng PENG, Cong LIANG, Jie LIU, Yunlu LIU, Ping LIU

**Affiliations:** 1 Department of Obstetrics and Gynecology, Nanfang Hospital, Southern Medical University, Guangzhou, China; 2 Department of Gynecology, Shenzhen People’s Hospital (The Second Clinical Medical College of Jinan University; The First Affiliated Hospital of Southern University of Science and Technology), Shenzhen, China

**Keywords:** Cervical cancer, cervical intraepithelial neoplasia, tRNA-derived small RNAs, vimentin, ZO-1

## Abstract

**Background/aim:**

Cervical cancer (CC) is a malignant gynecologic tumor. Small RNAs derived from tRNAs (tsRNAs) have been reported to play regulatory roles in tumor progression and suppression. However, the functional role of tsRNAs in CC remains largely unclear.

**Materials and methods:**

Small RNA sequencing was performed on normal, cervical intraepithelial neoplasia (CIN), and CC clinical samples to identify differentially expressed tsRNAs (DEtsRNAs). qRT-PCR was used to validate the selected DEtsRNAs. The effects of tsRNA-05020 on HeLa cell proliferation, apoptosis, and epithelial-to-mesenchymal transition were assessed.

**Results:**

Compared with the normal group, 215 tsRNAs were significantly differentially expressed in CIN tissues, of which 196 were upregulated and 19 were downregulated. A total of 184 DEtsRNAs were identified between CC and CIN tissues, including 81 upregulated and 103 downregulated in CC. In total, three candidate DEtsRNAs were validated, among which tsRNA-05020 exhibited the largest fold change and was significantly downregulated in CC compared with CIN. Target gene network analysis identified 278 putative target mRNAs of tsRNA-05020 in CC. Overexpression of tsRNA-05020 significantly inhibited HeLa cell proliferation and promoted apoptosis. Moreover, overexpression of tsRNA-05020 significantly reduced vimentin expression and increased ZO-1 expression.

**Conclusion:**

This study identifies a previously uncharacterized role of tsRNA-05020 in CC progression, expands the current understanding of tsRNA-associated regulatory networks, and suggests that tsRNA-05020 may serve as a potential molecular regulator in CC.

## 1. Introduction

Cervical intraepithelial neoplasia (CIN) is a well-established precursor of cervical cancer (CC) (Wang et al., 2022). Cervical cancer remains one of the leading threats to the health of women of reproductive age worldwide (Janicek and Averette, 2001). Persistent human papillomavirus (HPV) infection, influenced by factors such as high-risk sexual behavior, immunodeficiency, smoking, and multiparity, is the primary etiological factor associated with CIN and CC (Siegel et al., 2023). However, despite advances in screening strategies, the incidence of CC remains high because of suboptimal screening coverage and methodological limitations (Ortiz et al., 2021). The feasibility and therapeutic potential of CC-related molecular targeted therapies have been supported by several studies (Gao et al., 2019; Wei et al., 2020). The continuous identification and characterization of novel CC-related molecular regulators remain an urgent priority in current research. However, currently available molecular targeted therapies provide limited clinical benefit for patients.

Small RNAs derived from tRNAs (tsRNAs) are generated from precursor or mature tRNAs through specific cleavage events mediated by enzymes such as angiogenin and, in certain contexts, Dicer (Chen et al., 2021). These small RNAs have been implicated in stress responses, cellular signaling, and gene regulation, thereby contributing to cancer pathogenesis and progression. For instance, i-tRF-GlyGCC has been reported to improve risk stratification and predict unfavorable prognosis in patients with epithelial ovarian cancer (Panoutsopoulou et al., 2021). Evidence from a recent study showed that tRF-3022b reduces M2 macrophage polarization in colorectal cancer by interacting with LGALS1 and macrophage migration inhibitory factor (Lu et al., 2022). Additionally, tRFs have been proposed as novel diagnostic biomarkers in prostate cancer (Liu et al., 2023). Chen et al. (2022a) suggested a potential association between tsRNAs and epithelial-to-mesenchymal transition (EMT) in colorectal cancer. These findings highlight the multifaceted functional roles of tsRNAs in cancer biology. However, the roles of tsRNAs in CC remain insufficiently characterized, and their involvement in CIN has not yet been clearly defined. This study aims to elucidate the specific role of tsRNAs in CC and to characterize tsRNA expression profiles in both CIN and CC.

EMT plays a critical role in the progression and metastasis of CC. EMT-mediated cellular plasticity enhances the adaptability of cancer cells to the dynamic tumor microenvironment (Yang et al., 2022). Immune escape mediated by partial EMT and macrophage infiltration has been shown to promote lymph node metastasis in CC under lymph node metastasis associated suppressor (LNMAS) downregulation (Liao et al., 2022). EMT in CC has been reported to be regulated by miRNA-associated DNA methylation through the targeting of EZH2 (Chen et al., 2022). The malignant transformation of CC cells has also been closely associated with EMT (Peng et al., 2022). EMT has been shown to induce miR-31-3p-mediated chemoresistance in CC cells (Jing et al., 2019). In addition, the canonical EMT markers vimentin and ZO-1 have been reported to be significantly differentially expressed in CC.

Although the molecular mechanisms underlying CC have been increasingly elucidated, the progression of CIN to CC remains incompletely understood. This study aimed to characterize tsRNA expression profiles in CIN and CC using small RNA sequencing and to investigate the functional role of a key tsRNA in CC. These findings may provide valuable insights into the molecular mechanisms underlying CC progression.

## 2. Materials and methods

### 2.1. Clinical samples

A total of nine clinical samples were included for small RNA sequencing, comprising three paraneoplastic normal tissues, three CIN tissues, and three CC tissues. Detailed information regarding the clinical samples is provided in Table. Written informed consent was obtained from all patients prior to sample collection. This study was approved by the Ethics Committee of Nanfang Hospital, Southern Medical University.

### 2.2. HeLa cell culture and transfection

HeLa cells obtained from Procell (Wuhan, China) were used for subsequent in vitro experiments. HeLa cells were cultured in MEM medium (PM150410; Procell, Wuhan, China) supplemented with nonessential amino acids, 10% fetal bovine serum (FBS) (10099-141; GIBCO, Thermo Fisher Scientific, Waltham, MA, USA), and 1% penicillin–streptomycin at 37 °C in a humidified atmosphere containing 5% CO_2_. Trypsin used for cell passaging was purchased from Sangon Biotech (Shanghai, China). The cryopreservation solution consisted of 90% FBS and 10% dimethyl sulfoxide. Cells were suspended in the cryopreservation solution and stored at −80 °C until use.

Lipofectamine 2000 (1166019; Invitrogen, Carlsbad, CA, USA) was used to transfect HeLa cells with either a negative control (NC) or a tsRNA-05020 mimic. The sequences of the tsRNA-05020 mimic and NC (5′–3′) were as follows: tsRNA-05020 mimic,CGCCCGGCTAGCTCAGTCGGTAGAGCATGGGACTC; NC,UUCUCCGAACGUGUCACGUTTACGUGACACGUUCGGAGAATT (Shanghai Generay Biotech Co. Ltd., Shanghai, China). The culture medium was replaced with fresh medium 6 h after transfection, and the cells were incubated for an additional 24 h before subsequent experiments.

### 2.3. RNA extraction and quantitative real-time PCR

Total RNA was extracted from CC and CIN clinical tissue samples using TRIzol reagent (T9424; Sigma-Aldrich, St. Louis, MO, USA). RNA quality was assessed using spectrophotometry and agarose gel electrophoresis prior to reverse transcription. For reverse transcription, a 12 μL arbitrary primer system and a 12 μL gene-specific primer system were used. The 20 μL reverse transcription reaction mixture consisted of 4 μL of 5X buffer, 2 μL of dNTP mix, 1 μL of RNase inhibitor, 1 μL of reverse transcriptase, and 12 μL of primer mix. The reverse transcription conditions were as follows: 42 °C for 6 min, followed by 70 °C for 5 min. The synthesized cDNA was stored at −80 °C until further use. Quantitative real-time PCR (qRT-PCR) was performed using a 10 μL reaction mixture containing 5 μL of 2X master mix, 0.3 μL of forward primer (10 μM), 0.3 μL of reverse primer (10 μM), 3.4 μL of ddH_2_O, and 1 μL of cDNA. All reaction components were prepared on ice. The amplification conditions were as follows: 95 °C for 10 min (initial denaturation), followed by 45 cycles of 95 °C for 15 s and 60 °C for 60 s. Relative gene expression levels were normalized to U6 and calculated using the 2^-ΔΔCt^ method. Primer sequences are provided in Table S1.

### 2.4. Small RNA sequencing

Small RNA library preparation and sequencing were performed by MivectorBio (Shanghai, China). Briefly, 3′ and 5′ adapters were ligated to the RNA, followed by hybridization with a reverse primer. Subsequently, the ligated RNA products were reverse-transcribed into cDNA and amplified to construct cDNA libraries. Library quality was assessed using the Agilent 2100 Bioanalyzer (Agilent Technologies Inc., Santa Clara, CA, USA) according to the manufacturer’s instructions.

Samples were sequenced on the Illumina HiSeq 2500 platform using rapid run mode (Illumina, San Diego, CA, USA). Raw base call files were converted into FASTQ format. Adapter sequences were trimmed from raw reads using Cutadapt, and reads with low quality (Phred score < 20) or ambiguous bases were removed. Overall quality assessment of the raw sequencing data—including base quality distribution, GC content distribution, PCR duplication levels, and k-mer frequency—was performed using Fast-QC^1^ software. To eliminate interference from miRNAs and piRNAs, cleaned reads were aligned to miRBase^2^ and piRNAclusterDB 2.0^3^ with no mismatches allowed. Reads mapping to Rfam annotations were removed from further analysis. Identification and classification of tsRNAs were performed by mapping reads to tRFdb^4^ and MINTbase^5^ using default scoring parameters, followed by read counting.

Differential expression analysis of tsRNAs was conducted using the DESeq2 R package with raw counts as input. Significantly differentially expressed tsRNAs (DEtsRNAs) were defined as those with |log_2_ fold change| > 1 and an adjusted p value (Benjamini–Hochberg false discovery rate, [FDR]) < 0.05. Target mRNAs were predicted using miRanda and RNAhybrid for significantly different miRNAs, known piRNAs, and tsRNAs identified in the CC versus CIN and CIN versus normal comparisons (score ≥ 150 and energy < −20 kcal/mol). Only targets predicted by both algorithms were retained. Gene Ontology (GO) and pathway enrichment analyses of the predicted targets were performed, with FDR < 0.05 considered statistically significant.

### 2.5. CCK-8 assay

HeLa cells were cultured in medium supplemented with 10% FBS for 24 h. Subsequently, a single-cell suspension was prepared, and the cell density in each group was adjusted to 1 × 10^4^ cells/mL. Then, 10 μL of Cell Counting Kit-8 (CCK-8) solution was added to each well. Optical density values were measured using a microplate reader at 0, 24, 48, 72, and 96 h.

### 2.6. Flow cytometry

Flow cytometry was performed to evaluate apoptosis in HeLa cells following transfection with the tsRNA-05020 mimic. The Annexin V-FITC/PI apoptosis detection kit was purchased from Beyotime (C1062; Shanghai, China). Cells were resuspended in binding buffer and transferred into flow cytometry tubes, followed by staining with 1 × Annexin V-FITC and PI according to the manufacturer’s instructions. The final cell density was adjusted to 2–5 × 10^5^ cells/mL. A total of 5 μL of Annexin V-FITC were added to 195 μL of the cell suspension, gently mixed, and incubated at room temperature for 15 min in the dark. Subsequently, 190 μL of 1 × binding buffer and 10 μL of propidium iodide were added, and apoptosis was analyzed within 4 h. Flow cytometric analysis was performed using a FACSVerse flow cytometer (BD Biosciences, Franklin Lakes, NJ, USA).

### 2.7. Western blot analysis

HeLa cells were lysed on ice using RIPA lysis buffer (cat no. 89901; Thermo Fisher Scientific Inc., Waltham, MA, USA). The cell lysates were mixed with loading buffer, heated at 95 °C for 5 min, and immediately cooled on ice for 5 min. After centrifugation, the supernatant containing total cellular protein was collected for subsequent analysis. Protein samples were separated by sodium dodecyl sulfate–polyacrylamide gel electrophoresis (SDS–PAGE) and subsequently transferred onto polyvinylidene difluoride (PVDF) membranes (Millipore, Billerica, MA, USA). Membranes were stained with Ponceau S to verify protein transfer efficiency. Membranes were blocked with 5% skim milk in Tris-buffered saline containing 0.1% Tween-20 (TBST) at 4 °C overnight. Blocked PVDF membranes were incubated at room temperature for 3 h with primary antibodies against GAPDH (60004-1-Ig; Proteintech Group Inc., Rosemont, IL, USA), vimentin (ab92547; Abcam plc, Cambridge, UK), and ZO-1 (13663S; Cell Signaling Technology, Danvers, MA, USA). After washing with TBST, membranes were incubated with horseradish peroxidase (HRP)-conjugated goat antimouse IgG (ab205719; Abcam plc, Cambridge, UK) or goat antirabbit IgG (ab6721; Abcam plc, Cambridge, UK) secondary antibodies at room temperature for 1–2 h. Protein bands were visualized using a high-sensitivity enhanced chemiluminescence detection kit (32209; Thermo Fisher Scientific Inc., Waltham, MA, USA) and imaged using a chemiluminescence imaging system. Band intensities of target proteins were quantified using ImageJ software and normalized to the internal reference protein.

### 2.8. Statistical analysis

Continuous variables are presented as the mean ± standard deviation (SD). Statistical analyses were performed using GraphPad Prism version 8.0 (GraphPad Software, San Diego, CA, USA). Differences between two groups were analyzed using a two-tailed Student’s t-test. A p < 0.05 was considered statistically significant. Cytoscape software was used to construct the tsRNA–mRNA interaction network.

## 3. Results

### 3.1. Expression profiling of DEtsRNAs in cervical precancerous lesions

**Figure 1:**
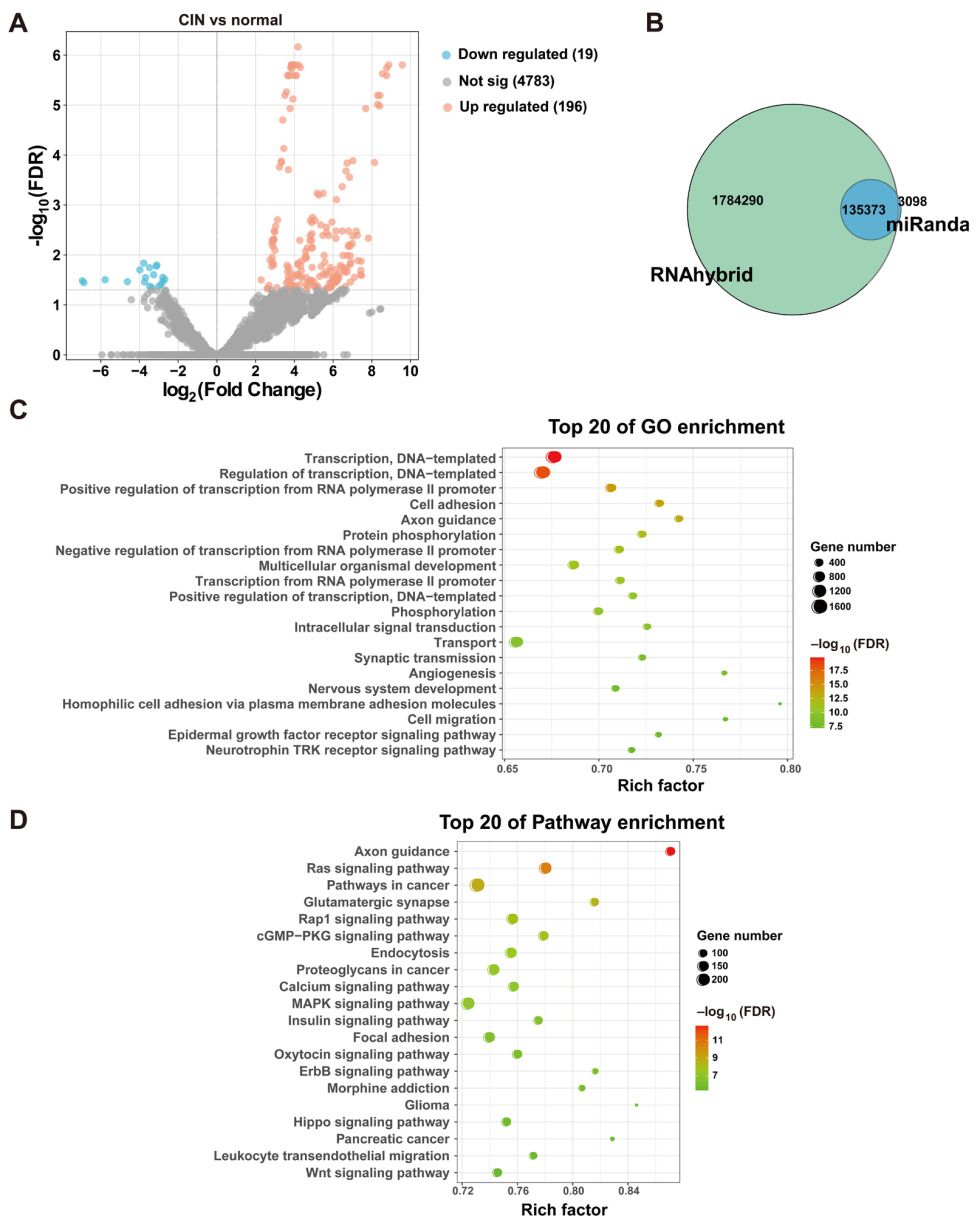
Expression profiling and functional enrichment analysis of DEtsRNAs in CIN. (A) Volcano plot showing DEtsRNAs in CIN compared with the normal group. Blue dots represent downregulated tsRNAs, whereas red dots represent upregulated tsRNAs in CIN. (B) Number of predicted target genes between the normal and CIN groups based on integrated analysis of RNAhybrid and miRanda databases. (C) GO enrichment analysis of predicted target genes of DEtsRNAs between the CIN and normal groups. (D) KEGG pathway enrichment analysis of predicted target genes of DEtsRNAs between the normal and CIN groups.

The development of CC represents a continuous pathological progression from precancerous lesions to invasive carcinoma. Early detection and timely treatment of CIN are critical strategies for reducing the incidence of CC (Cohen et al., 2019). However, significant gaps remain in the current understanding of the molecular mechanisms underlying CIN. A class of small noncoding RNAs known as tsRNAs has been reported in CC; however, their roles in CIN remain largely unexplored. To characterize tsRNA expression patterns in CIN, small RNA sequencing was performed to compare CIN samples with healthy control samples. After stringent quality control and preprocessing, a total of 215 DEtsRNAs were identified in the CIN group, including 196 significantly upregulated and 19 significantly downregulated tsRNAs (Figure 1A; Table S2). Subsequently, interaction analysis based on the RNAhybrid database^6^ and the miRanda database^7^ predicted 135,373 target genes associated with these DEtsRNAs (Figure 1B). GO and pathway enrichment analyses were subsequently performed for the predicted target genes of DEtsRNAs. GO and pathway enrichment analyses revealed significant enrichment in biological processes such as regulation of transcription, cell adhesion, protein phosphorylation, and cell migration, as well as in pathways including the Rap1, Hippo, MAPK, cGMP–PKG, and Wnt signaling pathways (Figures 1C and 1D). These findings indicate that DEtsRNAs are dysregulated in CIN and may contribute to CIN progression through the regulation of cell adhesion, migration, and protein phosphorylation.

### 3.2. Distinct expression profiles highlight the potential significance of DEtsRNAs in CIN and CC

**Figure 2:**
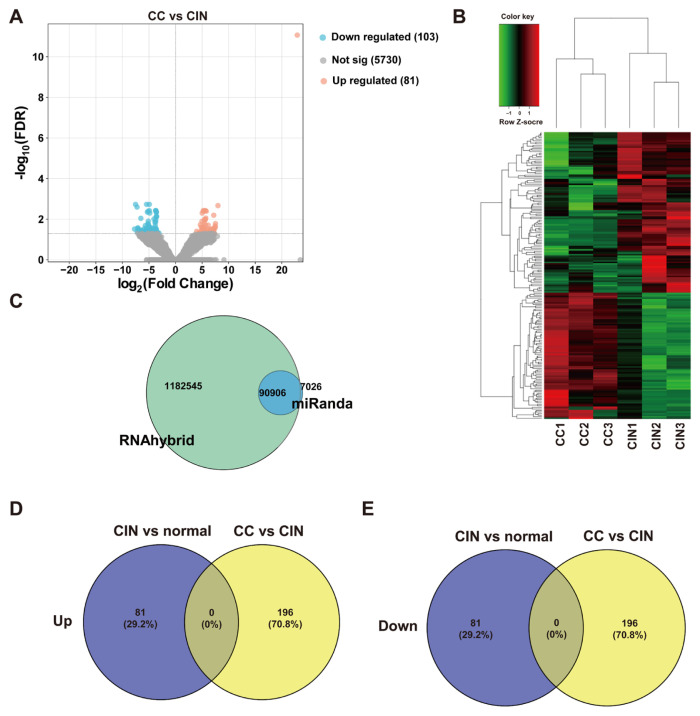
Expression profiling of DEtsRNAs in CC. (A) Volcano plot showing DEtsRNAs in CC compared with the CIN group. (B) Heatmap illustrating differential expression of DEtsRNAs in CC compared with the CIN group. (C) Venn diagram showing the number of predicted target genes in the CIN and CC groups. (D) Venn diagram showing the overlap of upregulated DEtsRNAs in CIN versus normal and CC versus CIN. (E) Venn diagram showing the overlap of downregulated DEtsRNAs in CIN versus normal and CC versus CIN.

**Figure 3:**
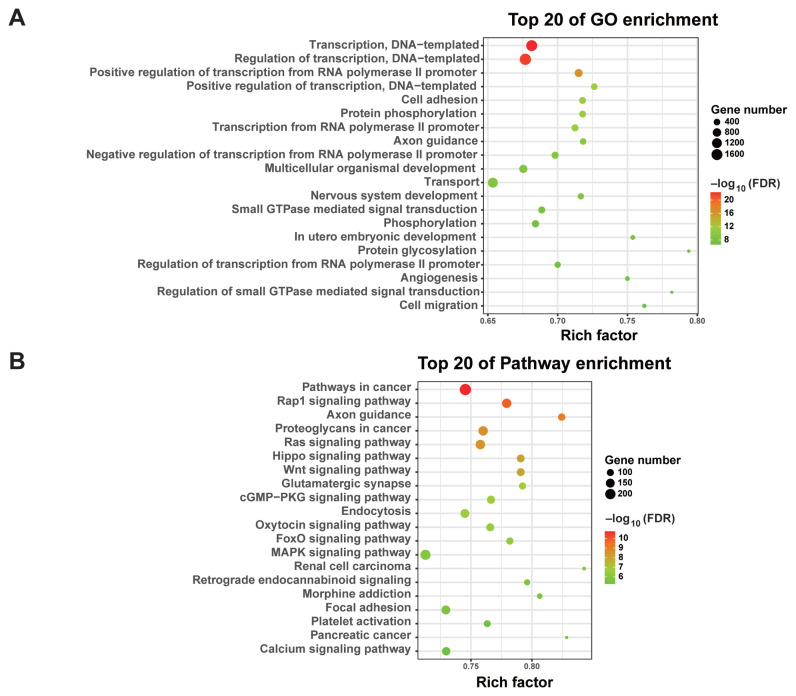
Functional enrichment analysis of DEtsRNAs between CIN and CC. (A) GO enrichment analysis of predicted target genes of DEtsRNAs between the CC and CIN groups. Panel A represents the CIN group, and Panel B represents the CC group. (B) KEGG pathway enrichment analysis of DEtsRNAs between the CIN and CC groups.

In addition to comparing CIN samples with healthy controls, small RNA sequencing was performed to identify differential tsRNA expression between CIN and CC samples. Compared with the CIN group, a total of 184 DEtsRNAs were identified in the CC group, including 81 significantly upregulated and 103 significantly downregulated tsRNAs (Figure 2A). Compared with CIN, tsRNAs in CC exhibited a more distinct differential expression pattern (Figure 2B). Furthermore, target prediction using the RNAhybrid and miRanda databases identified 90,906 putative target genes associated with these DEtsRNAs (Figure 2C). To determine whether DEtsRNAs identified in the CIN versus healthy and CC versus CIN comparisons were shared or distinct, an overlap analysis was performed using Venn diagrams. Notably, no overlapping DEtsRNAs were identified between the two comparisons in either the upregulated or downregulated groups (Figures 2D and 2E). This lack of overlap suggests that tsRNA expression profiles may undergo stage-specific alterations during disease progression. GO and pathway enrichment analyses of DEtsRNAs between CC and CIN revealed patterns similar to those observed in the CIN versus normal comparison. GO analysis revealed that DEtsRNAs in CC were primarily involved in cell adhesion, protein phosphorylation, and the positive regulation of transcription (Figure 3A). DEtsRNAs in CC were significantly enriched in the Rap1, Hippo, and Wnt signaling pathways (Figure 3B). Notably, compared with the CIN group, DEtsRNAs in the CC group were additionally enriched in homophilic cell adhesion via plasma membrane adhesion molecules, as well as in the epidermal growth factor receptor and ErbB signaling pathways (Figures 3A and 3B). These findings highlight functional differences in DEtsRNAs between CC and CIN and suggest the presence of stage-specific tsRNAs during the progression from CIN to CC.

### 3.3. Identification of DEtsRNAs in CIN and visualization of associated target gene networks

**Figure 4:**
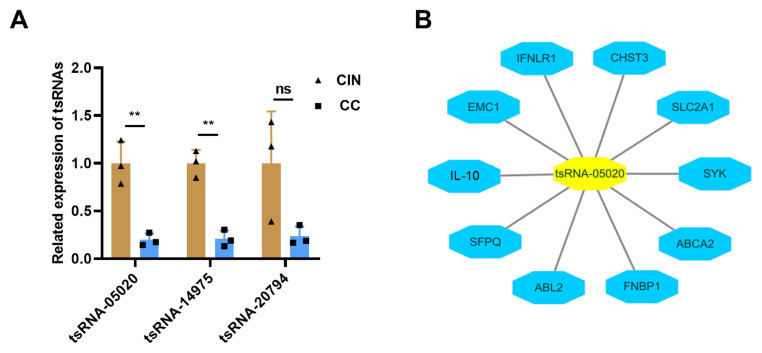
Identification of DEtsRNAs and visualization of target gene networks. (A) Expression levels of three candidate DEtsRNAs were validated in clinical tissues from the CC and CIN groups. **p < 0.01; ns indicates p > 0.05; n = 3. (B) Representative target genes of tsRNA-05020 and their interaction network are shown.

To validate the sequencing results, three tsRNAs exhibiting the largest fold changes and statistically significant differences were selected. As shown in Figure 4A, the expression levels of tsRNA-05020, tsRNA-14975, and tsRNA-20794 were downregulated in the CC group compared with the CIN group. Although both tsRNA-05020 and tsRNA-14975 were significantly downregulated, tsRNA-05020 exhibited a greater magnitude of downregulation. Compared with the CIN group, the difference in tsRNA-20794 expression in CC did not reach statistical significance (Figure 4A). Therefore, tsRNA-05020 was selected for subsequent functional verification studies. Based on predictions from the miRanda and RNAhybrid databases, 278 potential target genes of tsRNA-05020 were identified (Table S3). Given the large number of predicted targets, 10 well-characterized genes associated with tsRNA-05020 were selected for further analysis (ABCA2, ABL2, CHST3, EMC1, FNBP1, IFNLR1, IL10, SFPQ, SLC2A1, and SYK). The interaction network between tsRNA-05020 and these 10 target genes is shown in Figure 4B. For tsRNA-14975, target prediction analysis identified several cancer-related genes, including KRTAP10-2, RABL6, TRMU, CDK5, TONSL, TSGA13, CDK11B, AGPAT2, EML3, and HSCB (Figure S1A). Notably, CDK5 has been reported to participate in the regulation of chemoresistance in CC (Li et al., 2015), suggesting that tsRNAs may be involved in modulating treatment responses in CC. Similarly, target prediction analysis of tsRNA-20794 revealed a distinct regulatory network comprising CELA3B, CELA3A, APOBEC3C, MIER2, HOXB3, FZD1, ART1, LOC101929561, CUBN, and ZER1 (Figure S1B). Among these genes, APOBEC3C has been closely linked to HPV-associated cervical carcinogenesis (Ahasan et al., 2015), while HOXB3 has been reported to be aberrantly expressed in CC tissues (Liu et al., 2022), implicating tsRNAs in CC-related oncogenic processes. These target genes are associated with multiple oncogenic processes, including drug resistance, tumor progression, immune infiltration, and poor prognosis, suggesting that tsRNA-05020 may regulate CC through diverse target gene–mediated pathways.

### 3.4. Overexpression of tsRNA-05020 inhibits HeLa cell proliferation, promotes apoptosis, and regulates EMT

**Figure 5:**
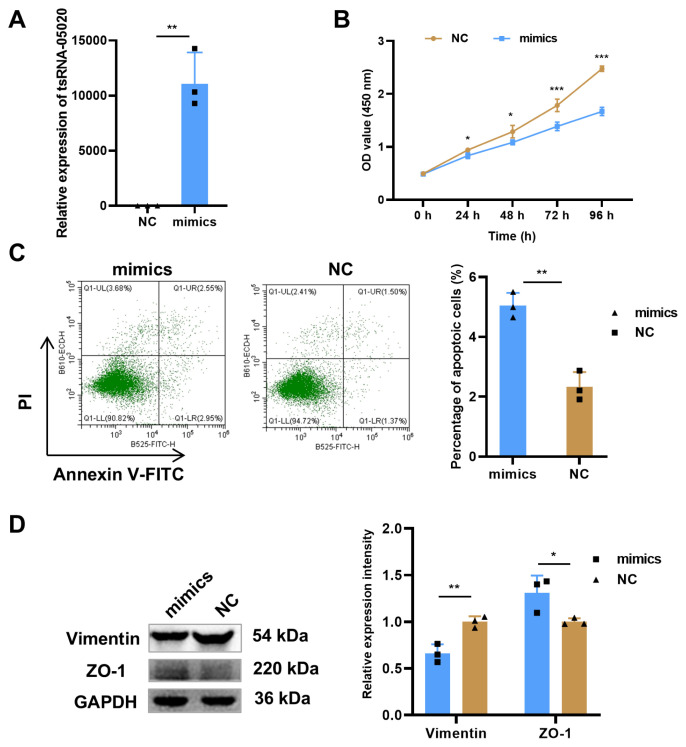
Overexpression of tsRNA-05020 inhibits HeLa cell proliferation, promotes apoptosis, and regulates vimentin and ZO-1 expression. (A) Overexpression efficiency of the tsRNA-05020 mimic was assessed by qRT-PCR (n = 3). (B) CCK-8 assay showing that tsRNA-05020 overexpression significantly inhibited HeLa cell proliferation (n = 6). (C) Flow cytometric analysis demonstrating that tsRNA-05020 overexpression significantly promoted apoptosis in HeLa cells (n = 3). (D) Western blot analysis demonstrating that tsRNA-05020 overexpression significantly downregulated vimentin and upregulated ZO-1 expression (n = 3). *p < 0.05; **p < 0.01; **p < 0.001.

To investigate the biological function of tsRNA-05020, HeLa cells were transfected with a tsRNA-05020 mimic to induce its overexpression. Efficient overexpression of tsRNA-05020 was confirmed by qRT-PCR (Figure 5A). CCK-8 assays and flow cytometry were performed to evaluate the effects of tsRNA-05020 overexpression on HeLa cell proliferation and apoptosis, respectively. Compared with the NC group, overexpression of tsRNA-05020 significantly inhibited HeLa cell proliferation and promoted apoptosis (Figures 5B and 5C). Subsequently, changes in EMT-related marker expression were examined, including the mesenchymal marker vimentin and the epithelial marker ZO-1. Vimentin and ZO-1 are well-characterized EMT-related proteins involved in cellular signaling and structural organization during disease progression (Ibrahim et al., 2020; Li et al., 2021). Overexpression of tsRNA-05020 significantly suppressed vimentin expression while enhancing ZO-1 expression (Figure 5D). These findings suggest that tsRNA-05020 may function as a potential suppressor of cervical cancer progression.

## 4. Discussion

Effective management of CIN represents a critical strategy for reducing the incidence of CC. The current treatment of CC mainly relies on surgery and chemotherapy. Although targeted molecular therapies have demonstrated promising therapeutic effects, their clinical application remains limited due to insufficient mechanistic and translational evidence. As emerging class of small noncoding RNAs, tsRNAs are involved in diverse biological processes, particularly in cancer development (Zhu et al., 2019). However, the roles of tsRNAs in CC remain insufficiently characterized. Small RNA sequencing identified 215 DEtsRNAs in CIN tissues compared with normal tissues and 184 DEtsRNAs in CC tissues compared with CIN tissues. Among these candidates, tsRNA-05020 exhibited the largest fold change and was selected for functional validation. Overexpression of tsRNA-05020 inhibited proliferation and promoted apoptosis in HeLa cells. The downregulation of vimentin and upregulation of ZO-1 further support a potential suppressive role of tsRNA-05020 in CC progression.

Among various classes of noncoding RNAs, tsRNAs are distinguished by their diverse mechanisms of action. A growing body of evidence indicates that these small noncoding RNAs function through competing endogenous RNA mechanisms, modulation of mRNA stability, regulation of ribosome biogenesis, and enhancement of translation. Numerous DEtsRNAs were identified, indicating a complex tsRNA expression landscape in CC. Although direct functional links between these additional DEtsRNAs and cervical cancer remain to be established, a resource is provided for future investigations of tsRNAs that may participate in tumor regulation. The molecular mechanisms underlying tsRNA function remain under investigation, with one proposed pathway being mediated by Argonaute (AGO) proteins. Interactions between tsRNAs and AGO1/AGO2 proteins were demonstrated using immunofluorescence assays (Haussecker et al., 2010). A candidate tsRNA, tsRNA-05020, was identified and was shown to significantly downregulate vimentin and upregulate ZO-1 protein expression. Based on these findings, it is hypothesized that tsRNA-05020 may regulate vimentin and ZO-1 expression through an AGO2-mediated mechanism. Vimentin has been reported to facilitate tumor progression (Satelli and Li, 2011), whereas ZO-1 has been characterized as a tumor suppressor (Zhang et al., 2019). This pattern is consistent with the present findings and provides supportive evidence for their biological plausibility. Moreover, vimentin is widely recognized as a hallmark marker of EMT (Usman et al., 2021), and ZO-1 has been reported to exert tumor-suppressive effects in EMT-associated cellular processes (Kim et al., 2023). Collectively, it is proposed that tsRNA-05020 may influence CC progression by modulating EMT through the regulation of vimentin and ZO-1. Additionally, significant inhibition of HeLa cell proliferation and enhancement of apoptosis were observed following tsRNA-05020 overexpression. These findings suggest that tsRNA-05020 may represent a potential therapeutic target in CC.

Rap1, a member of the Ras-related small GTPase superfamily, has been reported to coordinate cell localization, pathway activation, and gene regulation (Bondra and Rine, 2023; Ma et al., 2019; Singh et al., 2021). Pathway enrichment analysis demonstrated that the Rap1 signaling pathway was significantly enriched among CIN- and CC-associated DEtsRNAs. It has been reported that the Rap1 signaling pathway can be inhibited by berberine, leading to platelet activation and thrombosis (Wang et al., 2021a). CD73 has been reported to promote hepatocellular carcinoma progression and metastasis by inducing Rap1-mediated membrane localization of p110β and activation of the PI3K/AKT pathway (Ma et al., 2019). It has been reported that the Rap1 signaling pathway, in conjunction with the GREM1 signaling pathway, can be modulated by miR-205-5p to inhibit tumor metastasis in nonsmall cell carcinoma (Kan et al., 2022). Given its diverse roles in cell migration and tumor metastasis, Rap1 has been characterized as a key tumor-promoting regulator (Zhou et al., 2020). Collectively, these studies are consistent with the present GO and KEGG enrichment results, suggesting that DEtsRNAs may participate in CIN and CC progression through the Rap1 signaling pathway.

Notably, the Hippo signaling pathway was strongly associated with CC and was among the pathways significantly enriched by DEtsRNAs. The Hippo signaling pathway has been reported to promote radiation resistance in CC by regulating USP21-mediated deubiquitination of FOXM1 (Li et al., 2022). It has been reported that inactivation of the Hippo signaling pathway, resulting from interactions between NEK2 and the STRIPAK complex, facilitates CC cell proliferation (Zhang and Zheng, 2022). The Hippo–YAP signaling pathway has been identified as a central mechanism through which UM-6 induces autophagy and apoptosis in CC cells (Wang et al., 2021b). These observations are consistent with the present enrichment analysis, suggesting that the Hippo signaling pathway may represent a critical axis in the progression of CIN and CC.

Several tumor-related target genes associated with tsRNA-05020 were identified, including ABCA2, EMC1, SYK, IL10, and SLC2A1. ABCA2 contains two transmembrane domains, two nucleotide-binding domains, and a highly hydrophobic central loop region (Davis and Tew, 2018) and has been reported to be widely expressed in cancers and associated with resistance to multiple anticancer agents (Mack et al., 2008). EMC1 has been implicated in the progression of malignant tumors (Molina, 2014). SYK has been reported to exert context-dependent roles in tumor progression (Krisenko and Geahlen, 2015). IL-10–mediated immune modulation has been reported to influence inflammatory responses and regulatory T-cell (Treg) activity within the tumor microenvironment (Stewart et al., 2013). High expression of SLC2A1 has been associated with poorer survival outcomes in CC (Priego-Hernández et al., 2022). These findings provide indirect support for the predicted tsRNA-05020–associated target gene regulatory network. Collectively, indirect evidence is provided supporting the biological relevance of the predicted tsRNA-05020–associated regulatory network in CC.

Despite the novel insights obtained, several limitations should be acknowledged. First, the functional experiments were conducted only in vitro using a single HeLa cell line, which may not fully recapitulate the complexity of CIN and CC progression in vivo. Second, although potential target genes of tsRNA-05020 were identified through bioinformatic prediction, direct interactions and downstream signaling pathways require further experimental validation. Third, the clinical sample size was relatively limited, and additional cohorts are required to confirm the generalizability of these findings. Future investigations incorporating multiple cell lines, animal models, and larger patient cohorts will be essential for comprehensively assessing the biological and clinical relevance of tsRNA-05020.

In this study, the expression profile of 215 DEtsRNAs in CIN and 184 DEtsRNAs in CC tissues were characterized by small RNA sequencing. As a key DEtsRNA, tsRNA-05020 was shown to significantly inhibit proliferation and promote apoptosis in CC cells. Moreover, significant downregulation of the adhesion protein vimentin and upregulation of ZO-1 protein expression were observed following tsRNA-05020 overexpression. These data highlight the potential of tsRNA-05020 as a candidate molecular regulator and support the growing interest in tsRNA-mediated regulatory mechanisms in CC.

## Supplementary Information









## Figures and Tables

**Table:** Clinical characteristics of the enrolled samples.

Sample	Sample type	Date	Grade of CC	Other
1	Normal (tissue)	17 October 2022		
2	Normal (cell)			Primary cervical cancer cells directly isolated from patient specimens; cultured in vitro.
3	Normal (cell)			Primary cervical cancer cells directly isolated from patient specimens; cultured in vitro.
4	CPL (tissue)	15 September 2022	CIN2	Biopsy pathology: CIN1–2, HPV16 positive; conization performed; final pathology: CIN2.
5	CPL (tissue)	23 September 2022	CIN2–3	Biopsy pathology: CIN2; conization pathology: CIN2–3.
6	CPL (tissue)	14 October 2022	CIN1	Initial screening: CIN1; no biopsy at specimen collection; subsequent biopsy: focal CIN1.
7	CC (tissue)	9 October 2022		Biopsy performed; specimen contained both cellular and tissue components.
8	CC (tissue)	25 October 2022	Cervical well/moderately/poorly differentiated squamous cell carcinoma	Biopsy performed; no subsequent surgical resection.
9	CC (cell)			Primary cervical cancer cells directly isolated from patients samples.

Notes: CC, cervical cancer; CPL, cervical precancerous lesion; CIN, cervical intraepithelial neoplasia.

## Data Availability

The datasets generated and/or analyzed during the current study are available from the corresponding author upon reasonable request.

## Footnotes

1 Babraham Bioinformatics (2010). FastQC: A quality control tool for high throughput sequence data [online]. Website: http://www.bioinformatics.babraham.ac.uk/projects/fastqc/ [accessed 27 March 2026].

2 miRBase (2023). miRBase: the microRNA database [online]. Website: https://www.mirbase.org/ [accessed 27 March 2026].

3 Small RNA Group Mainz (2021). piRNAclusterDB: a database of piRNA clusters [online]. Website: https://www.smallrnagroup.uni-mainz.de/piRNAclusterDB [accessed 27 March 2026].

4 tRFdb (2014). tRFdb: a database for transfer RNA fragments [online]. Website: http://genome.bioch.virginia.edu/trfdb/ [accessed 27 March 2026].

5 MINTbase (2017). MINTbase: a database for tRNA-derived fragments [online]. Website: https://cm.jefferson.edu/MINTbase/ [accessed 27 March 2026].

6 RNAhybrid (2007). RNAhybrid: a tool for microRNA target prediction [online]. Website: http://bibiserv.cebitec.uni-bielefeld.de/rnahybrid [accessed 27 March 2026].

7 miRanda (2004). miRanda: a tool for microRNA target prediction [online]. Website: http://www.bioinformatics.com.cn/local_miranda_miRNA_target_prediction_120 [accessed 27 March 2026].
