# Supplementary figures and images for: Small RNA sequencing identifies tsRNA-05020 as a potential regulator of cervical cancer progression
Source: Turk J Biol. 2026 Feb 3;50(3):197–208. doi: 10.55730/1300-0152.2802 (PMC13398589; doi:10.55730/1300-0152.2802)

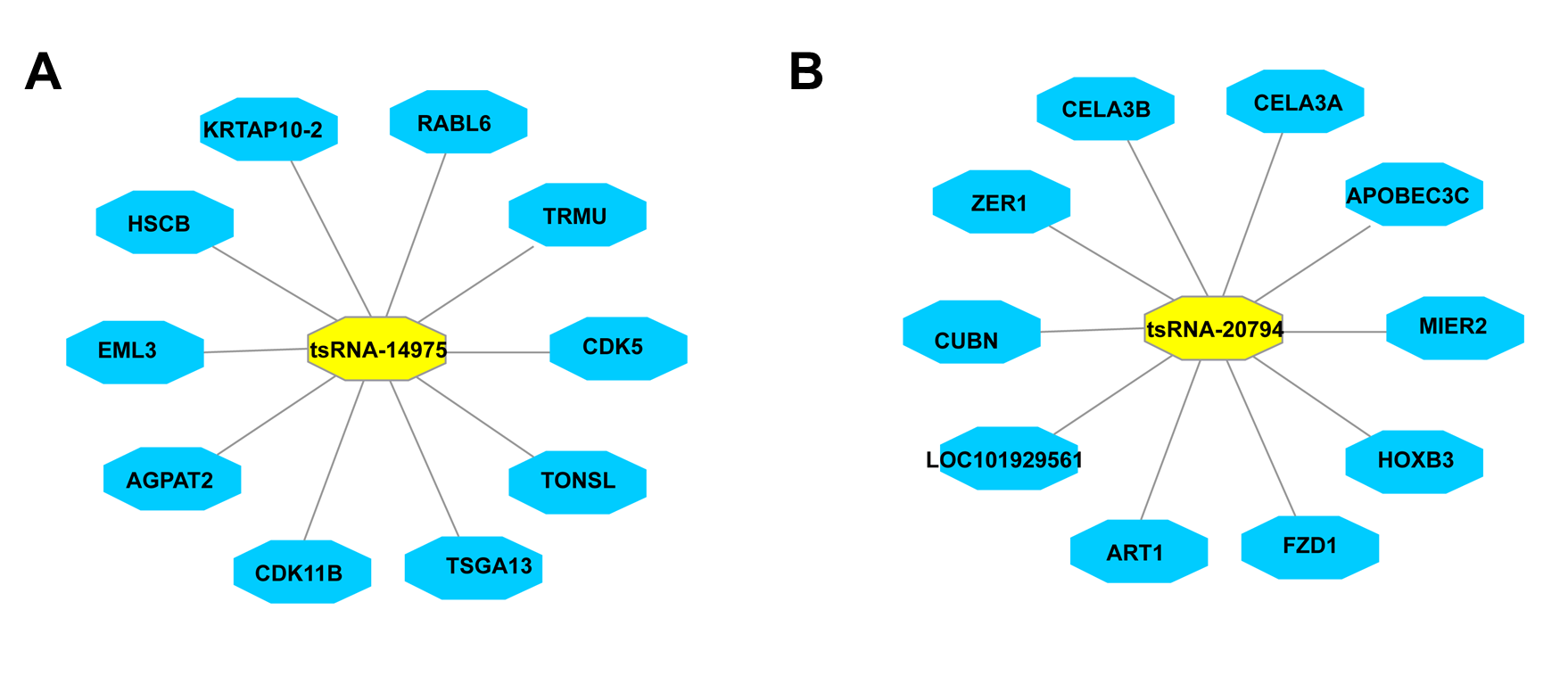

Supplement: Supplementary file 1 [file FigureS1.tif]
