# Supplementary material for: Small RNA sequencing identifies tsRNA-05020 as a potential regulator of cervical cancer progression
Source: Turk J Biol. 2026 Feb 3;50(3):197–208. doi: 10.55730/1300-0152.2802 (PMC13398589; doi:10.55730/1300-0152.2802)
Supplement: Supplementary file 2 [file SupplementalTable1.docx]

Supplementary Table 1 All the primer sequence in this study.

| **Gene** | **Primer sequence (5’ to 3’)** |
| --- | --- |
| Downstream random primer | AGTGCGTGTCGTGGAGTCG |
| U6-F | CGATACAGAGAAGATTAGCATGGC |
| U6-R | AACGCTTCACGAATTTGCGT |
| tsRNA-05020-F | CGCCCGGCTAGCTC |
| tsRNA-14975-F | GCCCGGCTAGCTCAG |
| tsRNA-20794-F | GCCGAGCGGTCTAAG |
| tsRNA-05020-RT | GTCGTATCCAGTGCGTGTCGTGGAGTCGGCAATTGCACTGGATACGACGAGTCCC |
| tsRNA-14975-RT | GTCGTATCCAGTGCGTGTCGTGGAGTCGGCAATTGCACTGGATACGACGAACGCA |
| tsRNA-20794-RT | GTCGTATCCAGTGCGTGTCGTGGAGTCGGCAATTGCACTGGATACGACGAACGCA |

*RT denotes specific reverse primers.
